# Review of Registries, Databases and Biobanks for Surveillance and Research on Mesothelioma

**DOI:** 10.3390/cancers18213441

**Published:** 2026-10-24

**Authors:** Sarita Prabhakaran, Tim Driscoll, Sue Barker, Geza Benke, Fraser Brims, Meredith Bryant, Elizabeth Chalker, Claire Cooke-Yarborough, Jodie Deakes, Justin Harvey, Susan Harden, Ewan Macfarlane, Kathleen Mahorney, Tom Watson, Phillip Wise, Amy Young, Ken Takahashi, Zivile Useckaite, David Roder, Karen Walker-Bone, Sonja Klebe

**Affiliations:** 1 Department of Anatomical Pathology, College of Medicine and Public Health, Flinders Health and Medical Research Institute, Flinders University, Adelaide, SA 5042, Australia; zivile.useckaite@flinders.edu.au (Z.U.); sonja.klebe@sa.gov.au (S.K.); 2 Sydney School of Public Health, University of Sydney, Sydney, NSW 2006, Australia; tim.driscoll@sydney.edu.au; 3 Australian Institute of Health and Welfare, Canberra, ACT 2601, Australia; sue.barker@aihw.gov.au (S.B.); claire.cooke-yarborough1@aihw.gov.au (C.C.-Y.); justin.harvey@aihw.gov.au (J.H.); tom.watson@aihw.gov.au (T.W.); amy.young@aihw.gov.au (A.Y.); 4 Monash Centre for Occupational and Environmental Health, Monash University, Melbourne, VIC 3004, Australia; geza.benke@monash.edu (G.B.); susan.harden@monash.edu (S.H.); ewan.macfarlane@monash.edu (E.M.); karen.walker-bone@monash.edu (K.W.-B.); 5 Curtin Medical School, Curtin University, Bentley, WA 6102, Australia; fraser.brims@curtin.edu.au; 6 Safe Work Australia, Canberra, ACT 2601, Australia; meredith.bryant@swa.gov.au; 7 National Centre for Epidemiology and Population Health, Australian National University, Acton, ACT 2600, Australia; elizabeth.chalker@act.gov.au (E.C.); phillip.wise@swa.gov.au (P.W.); 8 Asbestos and Silica Safety and Eradication Agency, Sydney, NSW 2001, Australia; jodie.deakes@asbestossafety.gov.au (J.D.); kathleen.mahoney@asbestossafety.gov.au (K.M.); 9 Asbestos Dust Diseases Research Institute, Concord, NSW 2139, Australia; ken.takahashi@uwa.edu.au; 10 School of Population and Global Health, University of Western Australia, Perth, WA 6009, Australia; 11 Department of Environmental Epidemiology of the Institute of Industrial Ecological, University of Occupational and Environmental Health, Kitakyushu City 807-8555, Japan; 12 Cancer Epidemiology and Population Health Research Group, Allied Health and Human Performance, University of South Australia, Adelaide, SA 5000, Australia; david.roder@unisa.edu.au; 13 South Australian Health and Medical Research Institute (SAHMRI), Adelaide, SA 5000, Australia; 14 Department of Anatomical Pathology, SA Pathology, Flinders Medical Centre, Bedford Park, SA 5042, Australia

**Keywords:** registry, mesothelioma, biobank, asbestos, bans, data recording

## Abstract

Mesothelioma is considered a signal tumour of asbestos exposure. Many countries have imposed asbestos bans or effectively no longer use asbestos, but a small number continue to import or mine it. Controls are generally much improved, with resulting plateauing or decreasing rates in many countries, but the effect of past exposures and ongoing exposure through in situ asbestos in buildings and environmental sources results in continued cases of mesothelioma globally. Improper disposal of asbestos is also an obstacle to managing and reducing asbestos exposure. Data on mesothelioma are recorded by mesothelioma registries, population-based cancer registries, healthcare databases, biobanks and online epidemiological data sources. Data from the various sources are significantly different and generally not directly comparable. Mesothelioma registries and population-based cancer registries are excellent resources for epidemiological and statistical data but are typically not linked to clinical data or biospecimen collections. Integrating biospecimen collections with linked clinical data creates rich real-world datasets that enhance research quality, enable data-driven discoveries, and support future translational research. The diagnosis of mesothelioma can be challenging, and data recording is not always accurate or complete across centres. Inconsistencies in data can affect epidemiological studies and undermine efforts to improve policies that affect individuals with mesothelioma and prevent further occurrence of mesothelioma. Linkage between mesothelioma registries, cancer registries, biobanks and other health-related databases will benefit researchers and allow access for further research initiatives. Closer collaboration between registries offers benefits for countries that lack diagnostic and healthcare infrastructure. Established mesothelioma registries can serve as valuable models for countries developing national surveillance systems by sharing expertise in case ascertainment, exposure assessment, registry governance and epidemiological methods. This study is a narrative review based on a non-systematic search of the published literature. This review of mesothelioma-related data resources and biospecimen collections serves as a foundation for enhancing collaboration and knowledge sharing in the fight against mesothelioma.

## Simple Summary

Mesothelioma is a rare cancer that is almost always linked to asbestos exposure. Information about people diagnosed with mesothelioma is collected through many different means, including disease registries, cancer registries, clinical databases, surveillance systems and biobanks. These differ in the type of information they collect and are not always connected, making it difficult to compare data or answer important research questions. This review summarises mesothelioma-related registries, databases and biobanks from around the world, highlighting their strengths, limitations and opportunities for collaboration. Better integration of clinical data, exposure histories and biospecimens has the potential to improve research, support patient care and strengthen efforts to prevent asbestos-related disease.

## 1. Introduction

Mesothelioma is a rare and aggressive cancer characterised by a long latent period between exposure and development of mesothelioma [1]. Asbestos exposure can lead to mesothelioma and other asbestos-associated diseases [2]. No safe threshold for asbestos exposure has been established [3]. In addition to asbestos exposure, inherited genetic predisposition has been implicated in increasing susceptibility to mesothelioma. The hereditary BAP1 tumour predisposition syndrome (TPDS) is characterised by a germline mutation of *BAP1* and associated with the development of mesothelioma and other neoplasms [4]. Since the diagnosis of mesothelioma can be challenging, differentiating benign mesothelial lesions and other malignancies from mesothelioma is important. Misdiagnosis may affect data recording and research findings.

**Figure 1:**
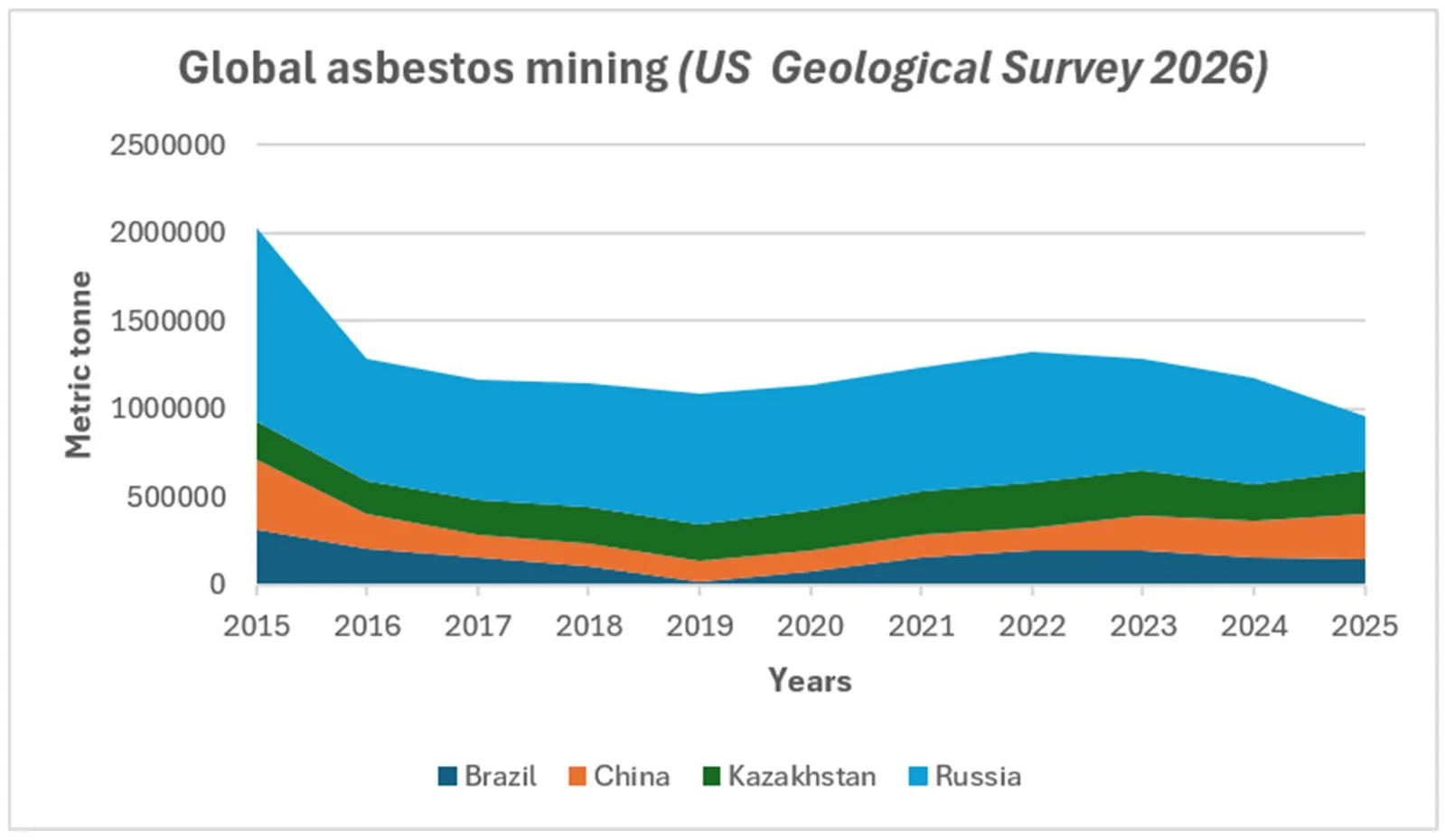
Global mining in metric tons (USGS 2026) [5]. Published with permission.

Since recognition of the health harms of asbestos, world production has reduced from an estimated 5.09 million metric tons in 1975 [2] to 960,000 metric tons in 2025 [5]. Countries that continue to mine asbestos include Brazil, China, Kazakhstan, and Russia (Figure 1).

Occupational standards to limit occupational asbestos exposure have been introduced in many countries, which may have lowered, but not eliminated, the risk of mesothelioma [6,7,8,9]. Currently, 73 countries have bans on asbestos [10], resulting in declining rates of mesothelioma [11,12]. A large proportion of countries with no bans in place are lower–middle-income and low-income countries [13]. Countries with higher socio-demographic indices (SDIs) have documented higher rates of mesothelioma, which may be due to the extensive use of asbestos in the past or relate to more complete recording [14].

### Types of Registries

Population-based cancer registries are designed to monitor the incidence, prevalence and stage of cancers, essential for the evaluation of cancer control programs [15] but do not routinely collect clinical data or treatment-related outcomes [16]. Hospital registries can have more administrative functions and support demographic and clinical care comparisons [17]. Clinical quality registries aid in monitoring and improving the quality of patient care and supporting epidemiological research [18].

Linkage between registries, insurance and healthcare databases helps improve the volume and validity of the data [19,20]. The functions of existing registries can be extended to support research studies, audits and recruitment of participants for clinical trials. In addition, they can provide opportunities for consumer and patient engagement and evaluation of health economics, thereby engaging more stakeholders, crucial for ongoing maintenance and support [21,22].

Biobanks are organised collections of well-characterised and annotated specimens including surgical samples, blood, DNA, RNA and other cell-derived components. Access to biobanked samples enables knowledge transfer of translational discoveries to the clinic [23]. Moreover, biobanks provide researchers with access to biospecimens and associated data that enable longitudinal cohort studies, biomarker discovery, molecular characterisation of tumours, and public health surveillance [24].

Mesothelioma registries aid in monitoring trends in mesothelioma, assess exposure histories and provide longitudinal data vital for epidemiological studies and future projections. Data entry may be linked to cancer registries, and in many countries with a history of asbestos use, mesothelioma is a notifiable disease.

This review aims to identify and compare worldwide mesothelioma registries, surveillance systems, clinical databases, cancer registries, biobanks and epidemiological data resources, evaluate their respective roles in surveillance, clinical care and research, and identify opportunities for integration, international collaboration and capacity building to advance mesothelioma prevention and improve patient outcomes.

A historical overview of global databases and research centres for asbestos and mesothelioma was last published in 1982 [25]. More recently, a 2020 review discussed the need for a national mesothelioma registry in the United States [26]. Since then, considerable advances have occurred in mesothelioma surveillance, registry development, clinical quality registries, biospecimen repositories, online epidemiological resources and international collaborations. The present review provides an updated and comprehensive comparison of worldwide resources for researchers, highlighting their complementary roles, opportunities for integration and linkage, and their contribution to research, clinical care and public health.

## 2. Methodology

### 2.1. Search Strategy

A literature search was undertaken to identify mesothelioma registries, surveillance systems, population-based cancer registries, clinical quality registries, disease-specific databases, biobanks and publicly accessible epidemiological data resources. PubMed and Google Scholar were searched from database inception to May 2026 using combinations of the following terms: mesothelioma, registry, cancer registry, asbestos, surveillance, database, biobank, biospecimen, occupational exposure, clinical quality registry, epidemiology and individual country names. A non-systematic search of the published literature was conducted to identify relevant resources and initiatives.

Relevant organisational websites were also reviewed, including national cancer registries, mesothelioma registries, surveillance agencies, international organisations and research networks. Reference lists of relevant publications were screened to identify additional resources.

Where publicly available information was incomplete, registry custodians, surveillance centres and biobank managers were contacted by email. This was particularly important for information on biospecimen holdings, clinical data linkage, governance arrangements and researcher access. Information obtained through personal communication was included where appropriate. This article was conducted as a narrative review intended to provide a broad overview of mesothelioma-related registries, databases and biobanks rather than as a formal systematic review.

### 2.2. Eligibility Criteria

Resources were included if they collected, stored, linked or reported mesothelioma-specific information, including biospecimens and data. Eligible resources included surveillance systems, population-based cancer registries, disease-specific registries, clinical quality registries, healthcare databases, biobanks and publicly accessible statistical platforms. Resources were excluded if they were not in English, did not contain mesothelioma-specific information, were correspondence articles or provided insufficient information.

## 3. Results

Data collected from the different resources are not always comparable. Differences in the purpose, governance, case ascertainment methods and data elements collected by individual registries and databases mean that comparisons should be interpreted in the context of each resource’s intended function rather than as measures of performance. A detailed comparison of major mesothelioma-specific registries and surveillance systems with sufficient publicly available information is presented in Supplementary Table S1. Resources that did not provide enough information for comparison are described in the text rather than included in the table.

### 3.1. Countries with Mesothelioma Registries

#### 3.1.1. Italy

In Italy, mesothelioma surveillance commenced in 1993 and was formalised nationally in 2002 with the establishment of the National Register of Malignant Mesothelioma (ReNaM). ReNaM has a number of regional operating centres (CORs) that actively search for cases of mesothelioma and obtain health information from hospital and pathology units. A trained interviewer then administers a questionnaire on occupational and residential history to individuals or their next of kin. Data from ReNaM is used for workers’ compensation from the Italian National Workers’ Compensation Authority (INAIL) [27]. ReNaM also records the type and sources of asbestos exposure and identifies unexpected sources of asbestos. Collaboration with other institutes has enhanced research opportunities and improved access for patients to clinical trials [22]. ReNaM offers important lessons from its extensive experience for countries with limited asbestos regulation and emerging mesothelioma surveillance systems [22].

#### 3.1.2. France

France has coordinated national systems for mesothelioma surveillance, combining epidemiological surveillance, expert pathological review, clinical data collection and biospecimen resources within interconnected national networks. Complementary programmes contribute different components of the surveillance pathway, collectively improving case ascertainment, diagnostic accuracy and research capability.

The Programme National de Surveillance du Mésothéliome (PNSM), established in 1998, forms the basis of population-based surveillance. It estimates mesothelioma incidence, monitoring temporal trends and investigating occupational, environmental and domestic asbestos exposure [28]. Following notification, trained investigators conduct structured face-to-face interviews with patients or, when necessary, their next of kin. Information collected includes complete occupational histories, residential history, schools attended, home renovation activities and other potential asbestos exposures. Industrial hygiene experts subsequently evaluate each exposure. Diagnostic confirmation and expert review are undertaken through the MESOPATH network, in which all suspected cases undergo expert pathological review before certification [29].

Clinical information from the MESONAT registry complements the PNSM, providing data on patient characteristics, treatment and outcomes for pleural mesothelioma [30], while RENAPE provides a national observational registry for rare peritoneal mesothelioma [31]. RENAPE is linked to a virtual tumour bank and facilitates multicentre clinical and translational research.

France has further expanded its national organisational infrastructure through NETMESO and MESOBANK, which together provide one of the world’s largest coordinated collections of mesothelioma biospecimens [29]. Frozen tissue, FFPE material, blood derivatives, digital pathology, molecular datasets and associated clinicopathological information are available within harmonised governance frameworks supporting biomarker discovery, translational studies and international collaboration.

A major development occurred in 2023 with the implementation of the National Mesothelioma Surveillance System (DNSM) [32]. As mesothelioma is now a notifiable disease throughout France, clinicians are legally required to notify cases to the Regional Health Agency, while all suspected diagnoses undergo expert review through MESOPATH before certification. The DNSM has created a coordinated “one-stop shop” for mesothelioma surveillance, strengthening public health monitoring, asbestos compensation processes and future research, aiming to achieve complete national case ascertainment.

#### 3.1.3. Australia

The Australian Mesothelioma Registry (AMR) was preceded by the Australian Mesothelioma Surveillance Program Register and formalised as a national mesothelioma registry in 2010. It was managed by the Cancer Institute New South Wales until 2017 and is currently run by the Australian Institute of Health and Welfare. Cancer registries in Australia are mandated to report mesothelioma to the AMR. Individuals can also self-notify to the AMR. Details on asbestos exposure are obtained from affected persons using a questionnaire and a telephone interview on residential and occupational histories, along with family history of mesothelioma. The AMR links to the National Death Index (NDI) annually to estimate survival [33].

Beginning in the 1960s, the Western Australian Mesothelioma Registry has been collecting data on mesothelioma from the state cancer registry and other healthcare facilities. Information on asbestos exposure is obtained from medical records, occupational and clinical histories, and, where available, interviews with patients or their next of kin. All exposures are coded, and diagnoses are confirmed by a committee consisting of a pathologist, an epidemiologist, an occupational physician and a respiratory physician. Mesothelioma is of particular interest to the Western Australian population because of the state’s crocidolite mine at Wittenoom, which operated from 1943 to 1966 [34].

#### 3.1.4. Germany

The German Mesothelioma Register was founded in 1973 and is located at the Ruhr University Bochum. It is an international reference centre for confirmation of the diagnosis of mesothelioma and analysis of asbestos fibre types and lung burden in exposed individuals [35]. In Germany, employers are required to participate in the statutory accident insurance system and maintain records of occupational exposure to carcinogenic and mutagenic substances. The German Social Accident Insurance (DGUV) maintains the Zentrale Expositions datenbank (ZED), a free centralised registry that records the nature, level, and duration of workplace exposures [36]. This database provides an important resource for the future recognition of occupational diseases.

#### 3.1.5. Belgium

The Asbestos Fund in Belgium was established in 2006 to certify and enable compensation for individuals with mesothelioma, confirm the diagnosis of diseases due to asbestos exposure and aid in compensation schemes. Currently, the Asbestos Fund is managed by Fedris, a governmental organisation. The Belgian Mesothelioma Registry (BMR) was established in 2010 at the University Hospital of Ghent, Belgium. The BMR assists with diagnostic confirmation of mesothelioma with the support of a panel of pathologists when specialist diagnostic opinion is required [20].

### 3.2. Other Surveillance Centres and Projects

The British Mesothelioma Register was established in 1967 to monitor mesothelioma mortality across Great Britain [37]. Cases were primarily identified through death certificates received from the Office of Population Censuses and Surveys and the General Register Office for Scotland, with cancer registrations, coroners’ reports, post-mortem reports, industrial death benefit records and reports from Health and Safety Executive regional medical advisers used to assess the completeness of case ascertainment. The register recorded demographic information, occupation, place of residence and anatomical site of mesothelioma but did not routinely collect detailed individual asbestos exposure histories.

Currently, the Health and Safety Executive (HSE) publishes national statistics on mesothelioma incidence, mortality and projected future deaths [38]. Mortality data continue to be derived from death certificates, while voluntary physician reporting is facilitated through a secure online reporting system [39].

In most European countries such as Austria, mesothelioma cases are recorded through mandatory occupational disease-reporting systems and population-based cancer registries rather than through dedicated mesothelioma registries. The absence of a mesothelioma-specific registry does not necessarily indicate that mesothelioma cases are not systematically recorded, as many countries capture these data through national cancer registration, statutory insurance, or occupational disease surveillance systems. These broader systems may lack the detailed exposure and clinical information typically collected by dedicated mesothelioma registries. The Nordic Occupational Cancer (NOCCA) project is a large study utilising pooled data from Sweden, Denmark, Norway, Finland and Iceland [40]. The NOCCA combines population census data (which includes information on occupation) and has developed country- and job-specific exposure matrices that provide estimates of exposure to specific carcinogens such as asbestos to determine risks and support preventive measures [41,42].

In Japan, the Environmental Restoration and Conservation Agency of Japan (ERCA) was established in 2004 to assist with compensation for individuals with asbestos-related diseases who are not covered under compensation legislation [43]. Information is collected from applicants requesting compensation using a structured questionnaire, together with supporting medical, occupational and residential records, to document occupational and environmental asbestos exposure histories and determine eligibility for compensation. As the database includes only individuals who apply for compensation, it does not represent all mesothelioma cases in Japan [43].

The Turkish Mesothelioma Working Group and the Public Health Institute initiated a national program for surveillance recording of mesothelioma (the TUNMES-EAECP). Cases are checked against entries in the national cancer registry system (MERNIS) for epidemiological studies [44].

South Africa has complementary resources for surveillance and research into asbestos-related diseases. The National Institute for Occupational Health (NIOH) undertakes national surveillance of mesothelioma and other occupational respiratory diseases and publishes annual reports describing disease trends [45]. In addition, autopsies are performed on deceased miners for compensation claims submitted by their families.

The NIOH also maintains the PATHAUT database, which contains information on more than 10,000 asbestos-related disease cases together with pathological diagnoses, occupational exposure information, mortality data and archived paraffin-embedded tissue [46]. Information is derived primarily from compensation-related autopsies and occupational pathology records rather than systematic population-based surveillance. As a result, coverage is greatest for workers entering the compensation system and may not fully represent all asbestos-related disease occurring within the broader community. Additional information is available through the Asbestos Relief Trust databases [47], which record demographic, diagnostic and asbestos exposure information for individuals seeking compensation, facilitating national and international collaborative research.

Legislation in Brazil mandates that employers in industries that use or previously used asbestos share details on health screening of employees and work history. A national database called Datamianto, established in 2022, collects personal information, asbestos exposure information and health records through physicians at hospitals in Brazil [48]. The data are used by the Ministry of Health for active surveillance and monitoring of exposed workers.

Thailand’s Health Data Centre (HDC), established in 2015, records mesothelioma and other asbestos-related diseases reported by hospitals under the Ministry of Public Health [49]. Reported cases are first reviewed by occupational health nurses to exclude errors such as incorrect coding. Occupational physicians then verify eligible cases by reviewing medical records. Occupational and environmental exposure histories are then obtained from patients, or their relatives when needed. However, the system is likely to underestimate the true number of cases because only correctly diagnosed cases and cases reported to the HDC are recorded.

Spain has a National Health Surveillance Programme for Workers Exposed to Asbestos, Programa Integral de Vigilancia de la Salud de los Trabajadores Expuestos al Amianto (PIVISTEA), which monitors the health of workers with past occupational asbestos exposure. To complement occupational surveillance, the Spanish Society of Pulmonology and Thoracic Surgery (SEPAR) established the voluntary Asbestos Respiratory Disease Registry (REPAS) in 2018 [50]. REPAS is a voluntary registry, and clinicians contribute demographic, occupational, clinical, respiratory function, radiological and follow-up information. However, geographical coverage is dependent on the distribution of collaborating clinicians.

The Korean Malignant Mesothelioma Surveillance system was active from 2001 to 2012. Members of the Cardiopulmonary study group of the Korean Society of Pathologists reported cases and included personal information, diagnostic results and other clinical information [51]. Currently, data for the Korean asbestos injury relief system is derived from surveys and questionnaires as well as health checks to assist with compensation due to asbestos exposure [52].

Occupational exposure registries in other countries provide opportunities for surveillance on the mechanism of asbestos exposure and to assess the risks of asbestos-related diseases. An example is the mandatory Ontario Asbestos Workers Register in Canada, which records information on the type and duration of asbestos exposure of employees and initiates health screening once an employee has completed 2000 h of exposure at work [53].

In Switzerland, the Swiss Laboratory for Particle Analysis in Tissues (Silag) maintains a database of clinical information and performs lung dust analysis on tissue samples from individuals with suspected asbestos exposure to quantify asbestos burden [54]. In the absence of a national mesothelioma registry, Silag represents an important resource for studying asbestos-related diseases; however, its utility is limited by incomplete demographic information, particularly occupational history and year of death.

### 3.3. Clinical Quality Registries and Databases

Within the Victorian Lung Cancer Registry is the Victorian Mesothelioma Outcomes Registry, which was launched in 2022 in Australia. It collects data on care and treatment for patients with mesothelioma in Victoria and conducts surveys that inform on quality of life and patients’ experiences. The registry data provide opportunities to identify variations in current practices and modify care in future to improve outcomes [55].

In addition to this state-based registry, Australia has two national clinical quality registries that record data on mesothelioma: the Australian Thoracic Cancers Longitudinal Cohort Study and Biobank (formerly AURORA) and the Australian and Aotearoa New Zealand Thoracic Clinical Quality Registry (ANZTHOR). These registries collect longitudinal clinical data on mesothelioma cases to inform clinical care and facilitate collaborative research between clinicians and researchers [56,57].

The European Society of Thoracic Surgeons (ESTS) database, established in 2001, was principally developed to assess quality standards for thoracic operations in Europe [58]. The database collects information from participating sites on individual mesothelioma cases, their diagnosis, treatment procedures and follow-up information that enables monitoring and auditing of clinical outcomes from thoracic surgery. Collaboration with the European Thoracic Oncology Platform (ETOP) Mesoscape research platform enables larger studies to understand and research the biology and optimal treatment for mesothelioma [59].

In the UK, the National Mesothelioma Audit (NMA) led by the Royal College of Physicians was established in 2016. It evaluates mesothelioma care and outcomes by collecting diagnostic, treatment, survival and patient experience information from sites across the UK to improve care and outcomes for individuals with mesothelioma [60].

The Japanese Joint Committee for Lung Cancer Registry (JJCLCR) is a clinical quality registry to better understand the epidemiology, management practices, and prognosis of pleural mesothelioma. It prospectively collects information on clinical, diagnostic, staging, treatment and survival data. The JJCLCR has contributed to the establishment of the staging system of lung cancer [61].

The International Society for the Study of Pleura and Peritoneum (ISSPP) in 2020 developed the PIPAC database to prospectively record clinical and treatment outcome data from patients undergoing Pressurised Intraperitoneal Aerosol Chemotherapy (PIPAC) procedures for peritoneal malignancies, including peritoneal mesothelioma [62], from more than 10 countries worldwide. Data are entered by participating ISSPP-accredited centres and include patient demographics, tumour characteristics, treatment details, perioperative complications, histological and radiological response, and longitudinal follow-up, enabling benchmarking, quality improvement and multicentre research.

The largest international database of pleural mesothelioma is the International Association for the Study of Lung Cancer (IASLC) database [63]. Through recommendations on refinements of previous iterations, the IASLC has helped make changes to the TNM staging system that have been adopted in the staging classification of the American Joint Committee on Cancer (AJCC) and Union for International Cancer Control (UICC). This global database includes mesothelioma diagnosed across multiple centres and geographical sites and has contributed to recommendations for revision of the ninth edition of the UICC/AJCC staging system [64].

### 3.4. Biobanks

**Figure 2:**
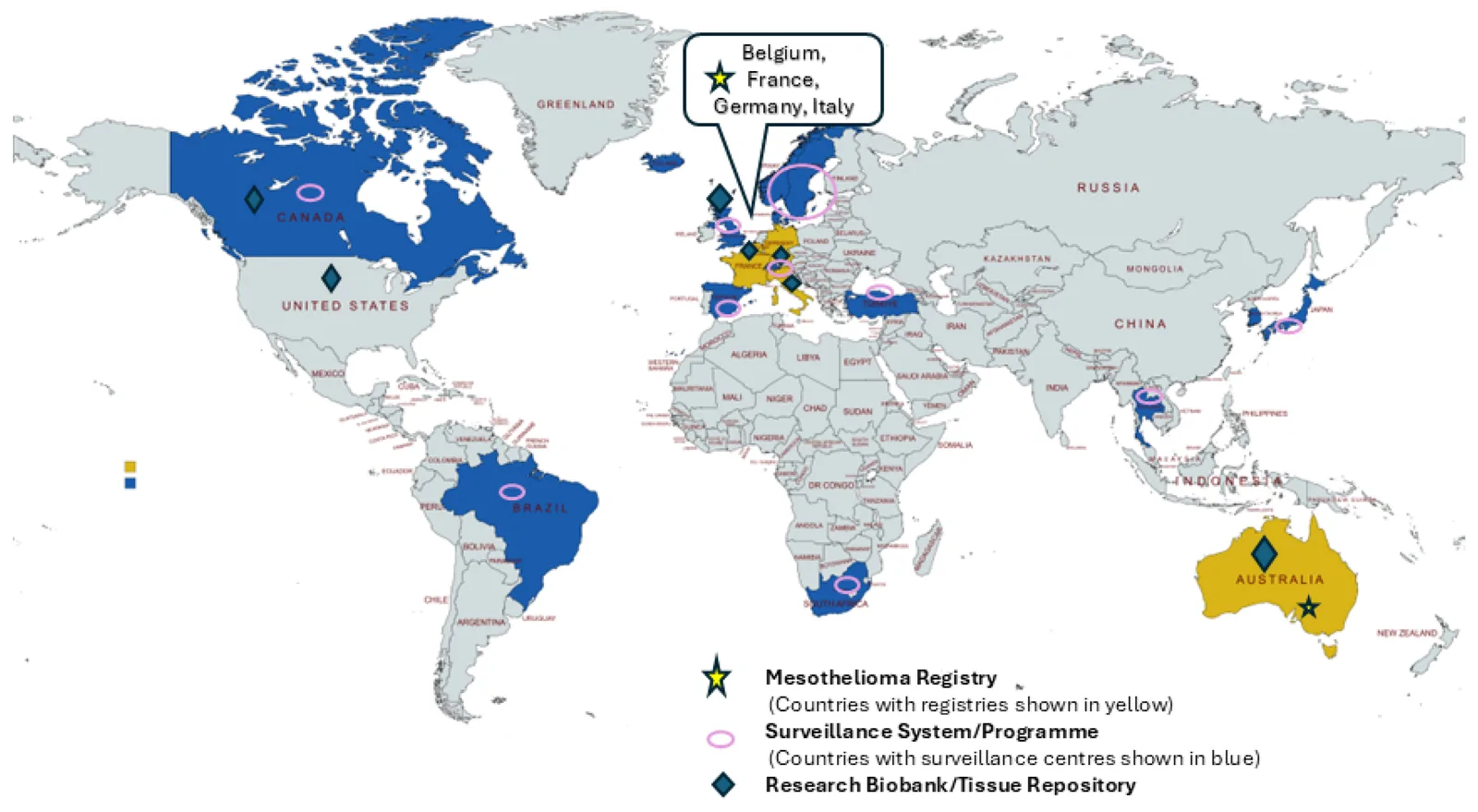
Global distribution of mesothelioma registries, surveillance programs, and biobanks.

Biobanks with mesothelioma samples may vary from small, dedicated collections of samples from individuals with mesothelioma to larger biobanks spread across regions with a broader range of biospecimens. Large, linked biobanks such as the Biobanking and Biomolecular Research Infrastructure–European Research Infrastructure Consortium (BBMRI-ERIC) have the infrastructure to connect researchers with required samples. The biobank of the ETOP Mesoscape has a centralised biobank that connects with labs within the network for specific projects. Examples of biobanks with mesothelioma samples can be found in Table 1. While many biobanks support collaborative research through the sharing of biospecimens and associated data, access is subject to institutional governance, scientific merit review, ethics approval, and the final approval of the individual biobank. The geographic distribution of mesothelioma registries, surveillance centres, and biobanks identified in this review is shown in Figure 2.

### 3.5. Online Databases

Several publicly accessible databases provide valuable statistical data that support global, regional, and national-level data on mesothelioma incidence, mortality, and trends over time. These include Globocan, NORDCAN, SEER Explorer and the Institute for Health Metrics and Evaluation (IHME). The IASLC database is a worldwide multisite collaborative collection of data on pleural mesothelioma, with data accessible to members. The Global Burden of Disease (GBD) platform by the Institute for Health Metrics and Evaluation (IHME) provides publicly available estimates of the burden of mesothelioma across countries and over time, with downloadable datasets and interactive visualisations (Table 2).

## 4. Discussion

Comprehensive data recording on the incidence, outcomes and survival of mesothelioma is crucial for advancing research and patient care [50]. Data collected from the different resources mentioned in this article differ significantly and are often poorly comparable. Deriving meaningful data requires further collaborative steps to ensure comparability. International collaboration for rare diseases such as mesothelioma through registries helps improve data quality by enabling robust analyses, which, in turn, should lead to improvements in patient care [65]. Mesothelioma registries are vital sources of epidemiological and statistical data. They mainly serve as surveillance units to monitor disease, and in some instances provide information on sources of asbestos that pose a risk to public health, and on latency of disease. Many mesothelioma registries and surveillance centres are not integrated with clinical datasets, so they do not include diagnostic procedure information or treatment outcomes. Also, mesothelioma registries predominantly focus on mesothelioma but not other asbestos-associated diseases such as pleural fibrosis, asbestosis, lung cancer, ovarian and laryngeal cancers, and perhaps cancers of the pharynx, stomach and colorectum [2]. Similarly, population-based cancer registries are equipped to provide statistical data for cancer control and policy development but are not routinely linked with biobanks or sources of patient-derived samples. Smaller datasets, especially from hospital-based systems and clinical registries, may not be able to provide statistical data on mesothelioma but can provide valuable insights into treatment response and prognostic risk factors of mesothelioma.

Delays in diagnosis and misclassification in the diagnosis of mesothelioma may also interfere with accurate and timely inclusion in healthcare and registry databases. This is more applicable to countries that lack adequate resources for specialist diagnostic facilities and data recording [66]. The rate of misclassification in diagnosis can range from 14% to 50% in developing countries, and mortality data are not always available, particularly from countries that still produce or use asbestos [66,67,68,69,70], resulting in underreporting of mesothelioma [71]. Factors that can hinder voluntary participation in data acquisition for mesothelioma registries include recall bias of past asbestos exposure and real or perceived potential legal ramifications of providing information to the registry.

Countries in which asbestos use has been restricted or banned show decreasing trends in the incidence of mesothelioma, though new cases continue to present due to the long latency of mesothelioma [72,73]. Countries still mining and/or using asbestos are expected to see potential increases in the rates of asbestos-related diseases [74,75].

Mesothelioma registries and dedicated databases have been key to novel research initiatives and supporting participant recruitment for clinical trials [22,29,31]. Record linkage of data between mesothelioma registries, cancer registries and other administrative data sources helps improve both the range and quality of collected data [19,20].

Countries with advanced technologies and knowledge of dealing with the epidemic of asbestos-related diseases should consider closer collaboration to share resources with countries that have only recently implemented bans or currently continue to use asbestos [76]. It is hoped that the advantages of established mesothelioma registries will be recognised and replicated globally to support efforts in asbestos-related disease prevention and patient care. Countries that currently do not have mesothelioma registries (such as Greece, Brazil, and Mongolia) have expressed an interest in developing such registries in their respective countries to raise awareness of the harms from asbestos, implement effective public health initiatives, improve surveillance, and enhance medical and support services for those affected [77,78,79]. Establishing registries in such settings would also support future international comparisons and collaborative research. Better documentation of asbestos exposure type, setting and duration can help identify preventable exposure sources and inform targeted public health interventions [80]. As mesothelioma surveillance systems continue to develop, the greatest gains are likely to come from linking epidemiological, clinical, exposure and biospecimen data within transparent governance frameworks.

A major strength of this review is the breadth of resources examined. In addition to mesothelioma registries and population-based cancer registries, this review includes surveillance systems, clinical quality registries, disease-specific databases, biobanks and publicly accessible epidemiological platforms. Presenting these resources within a single framework clarifies their different functions and highlights how they may contribute to surveillance, research and clinical practice.

Another strength is the inclusion of information obtained directly from registry custodians and biobank managers. This allowed incorporation of details on governance, biospecimen collections, data linkage and researcher access that are not consistently reported in publications or on institutional websites. This review also provides an updated synthesis of recent developments in mesothelioma surveillance, biobanking and international collaboration.

One of the limitations of this review is the lack of uniformity in available data from each resource. Some resources have extensive published documentation, whereas others are described mainly through institutional websites or personal communication. Registry structures, governance arrangements, specimen availability and data access procedures may also change over time, and some relevant resources may not have been identified despite broad searching. Finally, direct comparisons between registries should be made cautiously because their objectives, inclusion criteria, case ascertainment methods and data collection frameworks differ substantially.

## 5. Conclusions

Mesothelioma data are collected through a wide range of methods, including dedicated mesothelioma registries, cancer registries, clinical quality registries, surveillance programmes, research databases and biobanks. These systems differ in purpose, coverage, governance and data elements, and their outputs are not always directly comparable. No single resource captures all the information required for global mesothelioma surveillance, clinical care and research. Integration of comprehensive clinical data, biospecimen resources, multidisciplinary collaboration, and emerging technologies is essential to advance research, improve patient outcomes, and support mesothelioma prevention.

## 6. Future Directions

Future progress will depend on better linkage between epidemiological, clinical, exposure and biospecimen data. International collaboration, transparent governance and shared approaches to exposure assessment and diagnostic validation will be particularly important for countries developing new surveillance systems. Strengthening these connections will improve the quality of mesothelioma research, support patient care and contribute to more effective prevention of asbestos-related diseases.

## Figures and Tables

**Table 1:** Biobanks with mesothelioma samples.

Name	Location	Description	Specimens Collected
National Centre for Asbestos-Related Diseases (NCARD) biobank	Australia	National research centre-based biobank established in 1994	Tissue, blood pleural fluids, cell lines and urine
Asbestos and Dust Diseases Research Institute (ADDRI) biobank	Australia	National research institute-based biobank established in 2009	Fresh frozen tissue, FFPE tissue, pleural fluid, blood, primary cells and cell lines
Princess Margaret Cancer Biobank	Canada	University-based biorepository started in 2001	Frozen and FFPE tissue blocks
Thoracic Oncology Data and Biobank	Canada	University-based biospecimen collection	Tissue samples and blood products
Biobanking and BioMolecular Resources Research Infrastructure European Research Infrastructure Consortium	Europe	Pan-European network connecting national biobanks established in 2013	Tissues, FFPE, slides, effusions, blood products, radiology reports, clinical and treatment data
European Thoracic Oncology Platform (ETOP) Mesoscape	Europe	European decentralised biobank established in 2019	Tissue samples, clinical and treatment data
MESOBANK	France	National, established in 1998	Digitised slides, clinical and histologic database
Alessandria biobank	Italy	Hospital-based biorepository established in 1989	Frozen tissue, pleural fluid and blood products
CREST biorepository	Italy	National biorepository established in 1996	Tissue, pleural fluid, saliva, blood, serum and lymphocytes
Mesobank	UK	Biobank managed by an NHS Foundation Trust, established in 2012	FFPE tissue blocks, frozen tissue, pleural and peritoneal effusions, cell lines, DNA, RNA, serum and plasma
PREDICT-Meso Research Tissue Bank	UK	International research network-enabled tissue bank started in 2021	FFPE blocks, blood, plasma, serum, buffy coat, pleural fluid, DNA, RNA, protein, and exhaled-breath volatile compounds
National Mesothelioma Virtual Bank	USA	National virtual tissue bank established in 2006	FFPE tissue blocks, TMA, blood, serum, buffy coat, whole-slide images, and clinical and genomic data
Live-Cell Mesothelioma biobank	Zurich	National hospital-based biobank started in 2007	Cell lines

**Table 2:** Databases with statistical data on mesothelioma.

Database	Description	URL
Institute for Health Metrics and Evaluation (IHME)	The Global Burden of Disease based on the IHME provides free web-based tools for visualisation and publication of demographic data that includes mesothelioma.	https://www.healthdata.org/research-analysis/gbd (accessed on 14 October 2026)
Globocan	Part of the Global Cancer Observatory of the IARC. A free online database offers global population-based statistics on incidence and mortality from mesothelioma via Cancer Today and future projections on Cancer Tomorrow webpages.	https://gco.iarc.who.int/en/about-the-gco (accessed on 14 October 2026)
IASLC database	The largest global database on pleural mesothelioma. Data are used to refine AJCC/UICC tumour-staging systems.	https://www.iaslc.org/science-research/scientific-projects/iaslc-staging-project-lung-cancer-thymic-tumors-and (accessed on 14 October 2026)
Nordcan	A freely accessible online database with data on prevalence, incidence, mortality and survival for mesothelioma in Sweden, Norway, Greenland, Denmark Iceland, Finland and the Faroe Islands, derived from the Association of Nordic Cancer Registries	https://nordcan.iarc.fr/en (accessed on 14 October 2026)
Seer*Explorer	A free interactive website with cancer statistics from the Surveillance Epidemiology and End Results (SEER) Program across the US	https://seer.cancer.gov/statistics-network/explorer/application.html (accessed on 14 October 2026)

## Data Availability

Data collected during the current study are available from the corresponding author on reasonable request.

## Supplementary Materials

The following supporting information can be downloaded at https://www.mdpi.com/article/10.3390/cancers18213441/s1: Table S1. Major mesothelioma registries and databases, purpose, type of collections and research accessibility.
